# Supplementary material for: Impact of web accessibility on cognitive engagement in individuals without disabilities: Evidence from a psychophysiological study
Source: PLoS One. 2025 Jul 30;20(7):e0328552. doi: 10.1371/journal.pone.0328552 (PMC12310036; doi:10.1371/journal.pone.0328552)
Supplement: Attachment — Submitted filename: Response to Reviewers.pdf [file pone.0328552.s001.pdf]

Dear Reviewers and Editorial Board,

We would like to express our gratitude for your valuable comments and the opportunity to revise our manuscript according to your recommendations.

The manuscript has been thoroughly revised to address the reviewers' comments and enhance its technical and methodological quality.

We hope that the revised manuscript will be a suitable fit for the PLOS One Journal, and we would like to thank the reviewers for their support in achieving this.

For your convenience, we have highlighted the changes in the manuscript in *teal* color. The corresponding responses can be found in the right column of the table below.

Yours sincerely,

Merve Ekin

**Journal Requirements**	**Authors' Answers**
When submitting your revision, we need you to address these additional requirements.<br><br>1. Please ensure that your manuscript meets PLOS ONE's style requirements, including those for file naming. The PLOS ONE style templates can be found at https://journals.plos.org/plosone/s/file?id=wjVg/PLOSOne_formatting_sample_main_body.pdf and https://journals.plos.org/plosone/s/file?id=ba62/PLOSOne_formatting_sample_title_authors_affiliations.pdf	Thank you for addressing the formatting issues.<br><br>Based on the PLOS ONE style templates:<br><br>• We have changed "Figure" to "Fig" in the text.<br>• All figures were removed from the manuscript.<br>• All figures were uploaded in *.eps* version.<br>• Titles have been fixed.<br>• Tables have been updated based on the requirements.
2. Please note that PLOS ONE has specific guidelines on code sharing for submissions in which author-generated code underpins the findings in the manuscript. In these cases, we expect all author-generated code to be made available without restrictions upon publication of the work. Please review our guidelines at https://journals.plos.org/plosone/s/materials-and-software-sharing#loc-sharing-code and ensure that your code is shared in a way that follows best practice and facilitates reproducibility and reuse.	We fully agree with the importance of transparency and reproducibility in research. By PLOS ONE guidelines, we have shared author-generated code that supports the results and statistical power of our manuscript.<br><br>To improve clarity and usability for others, we have revised our script to load packages using standard libraries, namely "tidyverse", "afex", "emmeans", "WebPower", and "effectsize". This ensures that our code is accessible, easy to understand, and compatible with the R environments.<br><br>As suggested, we have added this code to the Zonedo repository (https://doi.org/10.5281/zenodo.15337424) with the previously shared data.
3. We note that the grant information you provided in the 'Funding Information' and 'Financial Disclosure' sections do not match.<br><br>When you resubmit, please ensure that you provide the correct grant numbers for the awards you received for your study in the 'Funding Information' section.	All funding information has been submitted accurately.

4. Please include a separate caption for each figure in your manuscript.	Captions have been added to each figure.
5. We note that Figure [xxxx] includes an image of a [patient / participant / in the study].<br>As per the PLOS ONE policy (http://journals.plos.org/plosone/s/submission-guidelines#loc-human-subjects-research) on papers that include identifying, or potentially identifying, information, the individual(s) or parent(s)/guardian(s) must be informed of the terms of the PLOS open-access (CC-BY) license and provide specific permission for publication of these details under the terms of this license. Please download the Consent Form for Publication in a PLOS Journal (http://journals.plos.org/plosone/s/file?id=8ce6/plos-consent-form-english.pdf). The signed consent form should not be submitted with the manuscript, but should be securely filed in the individual's case notes. Please amend the methods section and ethics statement of the manuscript to explicitly state that the patient/participant has provided consent for publication: "The individual in this manuscript has given written informed consent (as outlined in PLOS consent form) to publish these case details".<br><br>If you are unable to obtain consent from the subject of the photograph, you will need to remove the figure and any other textual identifying information or case descriptions for this individual.	The manuscript does not include a picture of the participant.
6. We are unable to open your Supporting Information file [paper.tex]. Please kindly revise as necessary and re-upload.	We have revised the file.

Reviewers' Comments	Authors' Answers
**Reviewer #1:**<br><br>Thank you for the opportunity to read and comment on the paper, Impact of Web Accessibility on Cognitive Engagement in Individuals Without Disabilities: Evidence from a Psychophysiological Study. This study examines how web accessibility features designed for individuals with low vision and cognitive impairments can benefit all users.<br><br>Using eye-tracking and heart rate variability measures, researchers analyzed the effects of these features on visual attention and cognitive processing in 20 participants without disabilities as they read news websites.<br><br>The results indicate that cognitive engagement typically declines over time but can be maintained with cognitive accessibility enhancements. Additionally, low vision accessibility features improve readability. Both self-reports and psychophysiological data confirm the advantages of incorporating these features, highlighting their broader implications for inclusive web design.<br><br>Overall, I found the paper to be very well written, organized and structured in a clear manner with a logical flow.<br><br>I appreciate the level of detail the authors provide in their hypotheses, methodological rigor, and statistical analysis plan. Their descriptions demonstrate rigor and transparency in their methods and analyses. I particularly found the analyses and results from the dynamics of visual attention to be a strong contribution from the study, as well as the mixed-methods and multimodal approach.	Thank you for your positive feedback and for finding our work relevant and interesting. We appreciate your recognition of our methodological rigor and the significance of our findings.
I only have some minor comments for the authors.<br><br>1. I have concerns regarding statistical power. I would like to see some explanation for how the sample size was chosen (e.g. a priori), and ideally, a power analysis. I found this lack of explanation surprising considering the level of statistical rigor in the rest of the text (it is not until the limitations section that the sample size is discussed).	Thank you for the comment.<br><br>We have added more information in the Participants section.<br><br>Answering directly to the comment on statistical power, we did not calculate *a priori* power analysis, however, we calculated it *post hoc*. The results of the post hoc power analysis are added to the manuscript for all statistically significant results. The power analysis results are discussed under the "Limitations and Future Directions" section, as suggested.

2. It is briefly addressed in the limitations section, but I have some questions and would like further explanation from the authors regarding the sample consisting of non-native English speakers being examined on English text, and their statement: "we have no reason to suspect that the effects of accessibility features would have a different impact on native speakers". I can think of several reasons why one might expect some different effects:<br><br>a. Studies have shown the use of subtitles, for example, differentially effects measures of realism, transportation, identification with characters, and other factors depending on if the subtitles are in ones own native language (e.g. Kruger, J. L., Doherty, S., & Soto-Sanfiel, M. T. (2017)<br><br>b. There are claims that second language reading is an interactive process, involving the interrelationship of cultural schemata and discourse structure – this could potentially have an impact on several of the measures included in this paper (see Barnitz)<br><br>c. There are even some specific findings on interpreting news media in non-native English speakers (Ward 2018), showing small but possibly relevant differences<br><br>The role that accessibility features might play in the above examples remains an open question – but I would like the authors to address this possibility or least better explain why they "have no reason to suspect" different impacts based on native language	Thank you for the suggestion.<br><br>The mentioned sentence has been deleted from the limitation.<br><br>Even though the participants were non-native English speakers, the study used a within-subjects design to focus on the changes in cognitive engagement between versions of the same websites with and without accessibility features.<br><br>Therefore, we have added more explanation to the Limitations and Future Directions section (Paragraph 1), in line with the Reviewer's comment.
**Reviewer #2:**<br><br>The Introduction:<br><br>Consider adding a brief discussion of the expected relationship between working memory capacity and cognitive engagement in the Introduction section to strengthen the hypotheses.	Thank you for this suggestion.<br><br>To clarify and support the hypothesis regarding working memory, we added more explanation to the Present Study section (Paragraph 2).<br>Thus, we establish a connection between working memory capacity and cognitive engagement, based on our purposes.
Definitions of key terms like "ambient attention," "focal attention," and "cognitive engagement" should be introduced earlier in the Introduction section in order to improve reader understanding.	Thank you for the comment.<br><br>The definition of cognitive engagement is already in the Introduction section.

	The definitions of ambient and focal attention have also been added to the Introduction (Paragraph 3).
The hypotheses are mentioned towards the end of the section. While they are clear, they could be better integrated into the narrative flow. Explaining how these hypotheses directly address the identified research gap would strengthen the introduction.<br><br>Related Works:<br><br>Related works provides a comprehensive literature review on web accessibility, cognitive engagement, and psychological measures. However, consider adding a section summarizing the research gap to improve coherence and align with the study's objectives. The authors should discuss how their study builds upon or challenges the findings of previous research.	Thank you for the suggestion.<br><br>To improve the manuscript's flow, the Present Study section was expanded by connecting the previous findings to our hypotheses, based on the originality of the present study (Paragraph 4).<br><br>To improve coherence and align with the study's objective, in the revised manuscript, we added a section summarizing the research gap.
Materials and methods:<br><br>The sample size (20 participants) is small, limiting generalizability. Provide a stronger justification for this sample size or address its limitations directly.	Thank you for the comment.<br><br>We have added more information in the Participants section.<br><br>Answering directly to the comment on sample size: We did not calculate *a priori* power analysis, however, we calculated it *post hoc*. The results of the post hoc power analysis are added to the manuscript for all statistically significant results. The power analysis results are discussed under the "Limitations and Future Directions" section, as suggested.
The authors should discuss if the use of non-native English speakers interacting with English website could bring a variability in the results.<br><br>Also, the description of participant characteristics could include more demographic details (e.g., educational background, cultural factors) to clarify sample diversity.	This use of the non-native English speaker was discussed in the Limitations and Future Directions section (Paragraph 1).<br><br>Thank you for your suggestion. Unfortunately, we do not have access to additional demographic information, such as participant education. We recognize the importance of such data and will consider including it in future studies.
The time allocated for interacting with each website version (60 seconds) and answering questions (45 seconds for comprehension, 15 seconds for readability and understandability) seems relatively short and the authors should justify this duration and discuss any potential effects on the results.	In line with the Reviewer's comment, we have added the following sentence and more explanation to the "Procedure" (Paragraph 2) section:<br><br>"The main reason was to avoid fatigue effects and time overload when considering the whole experiment. Previous studies using eye-tracking in web contexts have also used similar durations to assess attention and user engagement

	(Djamasbi, 2010; Shrestha, 2008)."<br><br>Djamasbi, S., Siegel, M., & Tullis, T. (2010). Generation Y, web design, and eye tracking. International Journal of Human-Computer Studies, 68(5), 307–323. https://doi.org/10.1016/j.ijhcs.2009.12.006<br><br>Shrestha, S., & Owens, J. (2008). Eye movement analysis of text-based web page layouts. Proceedings of the Human Factors and Ergonomics Society Annual Meeting, 52(19), 1510–1514. https://doi.org/10.1177/154193120805201916<br><br>The explanations were also added to the "Limitations and Future Directions" (Paragraph 4) section.
Results and discussion:<br><br>Results are presented logically with appropriate use of visual aids and statistical tests. While the results are presented clearly, the interpretation could be more detailed. The discussion of unexpected findings, such as the lack of significant differences in reading comprehension, could be expanded. The authors should speculate on potential reasons and implications more thoroughly.	Thank you for the suggestion.<br><br>The lack of significant differences in reading comprehension is discussed in more detail in the "Discussion/The Effects of Low Vision Accessibility Enhancements on Readability and the Ease of Cognitive Processing (Hypothesis 2)" section (Paragraph 2).
Other shortcomings:<br><br>The text lacks references to images.<br><br>The authors did not sufficiently address the assumptions of using the analysis of covariance.	 <br>All images have been referenced in the necessary parts of the manuscript.<br><br>Working memory capacity is used as a covariate factor in this study. This information and the reasons for it have been explained in detail in the "Present Study" section (Paragraph 2).
